# Use of the Xpert® MTB/RIF assay for diagnosing pulmonary tuberculosis comorbidity and multidrug-resistant TB in obstetrics and gynaecology inpatient wards at the University Teaching Hospital, Lusaka, Zambia

**DOI:** 10.1111/tmi.12145

**Published:** 2013-07-03

**Authors:** Matthew Bates, Yusuf Ahmed, Lophina Chilukutu, John Tembo, Busiku Cheelo, Sylvester Sinyangwe, Nathan Kapata, Markus Maeurer, Justin O'Grady, Peter Mwaba, Alimuddin Zumla

**Affiliations:** 1 Center for Clinical Microbiology, University CollegeLondon, UK; 2 University of Zambia and University College London Medical School Research and Training ProgrammeLusaka, Zambia; 3 Department of Obstetrics and Gynaecology, University Teaching HospitalLusaka, Zambia; 4 Department of Paediatrics and Child Health, University Teaching HospitalLusaka, Zambia; 5 National Tuberculosis Control Programme, Ministry of HealthLusaka, Zambia; 6 Department of Tumour Immunology and Microbiology, Karolinska InstituteStockholm, Sweden; 7 Ministry of HealthLusaka, Zambia

**Keywords:** tuberculosis, obstetric, gynaecology, maternal, women, Xpert® MTB/RIF assay, sensitivity, specificity, multidrug-resistant TB, MDR-TB, sub-Saharan Africa

## Abstract

**OBJECTIVES:**

In high-tuberculosis (TB)-endemic countries, comorbidity of pulmonary TB in hospitalised patients with non-communicable diseases is well documented. In this study, we evaluated the use of the Xpert® MTB/RIF assay for the detection of concomitant pulmonary TB in patients admitted to the University Teaching Hospital, Lusaka, Zambia, with a primary obstetric or gynaecological condition.

**METHODS:**

The Study population were inpatients admitted with a primary obstetric or gynaecological problem who had a concomitant cough and were able to expectorate a sputum sample. Sputum samples from 94 patients were analysed for the presence of *Mycobacterium tuberculosis* (*M.tb*) by standard smear microscopy, MGIT culture, MGIT drug-susceptibility testing (DST) and the Xpert® MTB/RIF assay. The sensitivity and specificity of the Xpert® MTB/RIF assay were evaluated against the culture gold standard.

**RESULTS:**

Twenty-six of 94 (27.7%) patients had culture-confirmed pulmonary TB. The Xpert® MTB/RIF assay had a sensitivity of 80.8% [95% CI: 60.0–92.7%]) compared against MGIT culture. The Xpert® MTB/RIF assay was more sensitive than sputum smear microscopy (21/26 (80.8%) *vs*. 13/26 (50.0%), *P* = 0.02) and detected an additional eight culture-confirmed cases. Culture DST analysis identified two monoresistant *M.tb* strains: one resistant to rifampicin (rifampicin sensitive by the Xpert® MTB/RIF assay) and one to ethambutol. HIV infection was linked with a 3-fold increase in risk of TB, accounting for 87.5% (21/24) of TB cases. 50% of cases presented as comorbidities with other communicable diseases (CDs) and non-communicable diseases (NCDs).

**CONCLUSIONS:**

As an alternative to sputum microscopy, the Xpert® MTB/RIF assay provides a sensitive, specific and rapid method for the diagnosis of pulmonary TB in obstetric or gynaecological inpatients. Pulmonary TB is an important cause of concomitant comorbidity to the obstetric or gynaecological condition necessitating admission. TB and HIV comorbidities with other communicable and non-communicable diseases were also common. More proactive screening for TB comorbidity is required in obstetric and gynaecological wards.

## Introduction

WHO estimates that tuberculosis (TB) killed 500 000 women in 2011, of which 200 000 cases were associated with HIV co-infection (WHO 2012). In sub-Saharan Africa (SSA), TB causes 15–34% of non-obstetric maternal deaths (Ahmed *et al*. 1999; Khan *et al*. 2001; Menendez *et al*. 2008; Black *et al*. 2009). African women of child-bearing age carry the highest HIV/TB codisease burden (WHO 2011a,2011b). Maternal and childhood TB are epidemiologically linked (Batra *et al*. 2012), and there is a close association between maternal TB and post-partum infant morbidity and mortality in children born to both HIV-infected (Cotton *et al*. 2008; Gupta *et al*. 2011) and HIV-uninfected women (Tripathy 2003; Lin & Chen 2010). In HIV-infected women, multivariate analysis has shown that maternal TB infection was independently linked with increased risk of mother-to-child transmission of HIV (Gupta *et al*. 2011). The risk of TB is 24-fold higher in HIV-infected infants (Hesseling *et al*. 2009) who are at increased risk of TB-associated mortality (Pillay *et al*. 2001). Pregnancy is an immunosuppressed state and a risk factor for developing active TB (McNerney *et al*. 2012).

Tuberculosis comorbidity with other communicable diseases and non-communicable disorders is well described, and there are calls for more proactive screening for TB at all points of care (Bates *et al*. 2012). TB comorbidity in women requiring obstetric or gynaecological inpatient care in high TB and HIV endemic countries may be easily overlooked because the focus of the admitting physician is on the primary obstetric or gynaecological reason necessitating admission. In addition, non-specific symptoms of active TB such as fatigue or night sweats are commonly associated with pregnancy, during and after childbirth, or in chronic gynaecological infectious or non-communicable diseases (Chang *et al*. 2012). Furthermore, the low sensitivity of sputum smear microscopy and the operational constraints of mycobacterial culture mean that not all TB cases are identified (McNerney *et al*. 2012). Several new technologies for the rapid diagnosis of TB are now commercially available such as the Xpert® MTB/RIF assay (Lawn *et al*. 2013). In this study, we evaluated the use of the Xpert® MTB/RIF assay for the detection of concomitant pulmonary TB in patients admitted to the University Teaching Hospital, Lusaka, Zambia, with a primary obstetric or gynaecological disorder.

## Materials and methods

### Ethics approval

This study was approved by the Biomedical Research Ethics Review Committee of the University of Zambia, School of Medicine, Lusaka. All study participants gave written informed consent, and the study was conducted in full accordance with all the ethics committee's guidelines.

### Design and aims

This was a descriptive, prospective study to evaluate the use of the Xpert® MTB/RIF assay for the detection of concomitant or undiagnosed pulmonary TB and drug-resistant TB in patients admitted primarily for an obstetric or gynaecological disorder to the University Teaching Hospital, Lusaka, Zambia.

### Patient recruitment

Patients admitted during the previous 24 h to the obstetrics or gynaecology wards were eligible for recruitment. Admission criteria were patients who had a cough and could produce a sputum sample unaided (without induction). Patients already on TB treatment were excluded. Informed consent was obtained from all the study participants. Clinical details of all the participants including the diagnosis that necessitated hospital admission were recorded.

### Definitions

*Smear positive* indicates presence of acid-fast bacilli (AFBs) in at least one sputum specimen on sputum smear microscopy. *Smear negative* means absence of AFBs in sputum specimens on sputum smear microscopy. *Culture positive* refers to a positive MGIT culture with a positive TBcID confirmatory test result and negative blood agar test. *Culture negative* denotes a negative MGIT culture or positive MGIT culture with a negative TBcID confirmation test result.

### Sample collection and laboratory processing

Patients with a cough, having given their consent, were requested to provide up to three sputum samples (spot-morning-spot). This was supervised by dedicated clinical staff in the wards. Sample decontamination, fluorescent smear microscopy, MGIT culture and phenotypic drug-susceptibility testing were performed as described previously (O'Grady *et al*. 2012).

### Xpert© MTB/RIF assay

0.5 ml of concentrated decontaminated sputum was added to a 15-ml Falcon tube in a 1:3 ratio with the sample reagent (0.5 ml of sputum sample to 1.5 ml of sample reagent), and the resulting mixture added to the Xpert® MTB/RIF assay cartridge and then run in the machine in accordance with the manufacturers guidelines (Lawn *et al*. 2013). ‘Error’ results were repeated.

### Data analysis

Two dedicated laboratory personnel blinded to all clinical recruitment, and sample labelling data processed all samples. Clinical and laboratory data were compiled in a database using Epidata software. Analysis was undertaken using SPSS version 18 (IBM, Armonk, NY, USA). The sensitivity, specificity and predictive values of Xpert® MTB/RIF assay were calculated with 95% confidence intervals (CI) and compared with smear microscopy using Pearson chi-squared test. Risk factors were evaluated by multivariate binary logistic regression.

## Results

### Study group

**Figure 1:**
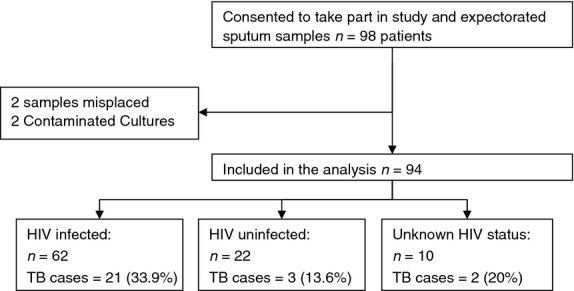
Recruitment flow diagram detailing TB and HIV status.

Ninety-eight inpatients consented to take part in the study and provided sputum for microbiological TB analysis. Four patients were excluded from the analysis (two specimens misplaced, and two cultures were contaminated), and so, the results from a total of 94 patients were analysed (Figure 1). 67.0% (63/94) of recruited women were pregnant or <6 weeks post-natal when recruited, and 33.0% (31/94) were not pregnant or post-natal. Median age was 28 years (IQR: 24–32). HIV status was available for 84 patients, and HIV prevalence was 73.8%. 11 patients had a history of TB treatment, 10 of whom were HIV positive and one was of unknown HIV status (Table 1). The primary admission diagnosis given by the attending physician was recorded. Suspicion of TB alone was noted for 25.5% (24/94) of patients, with an additional 30.9% (29/94) having an admission diagnosis of pneumonia and suspected TB. The remaining 41 patients presented with a range of admission diagnoses including 29.8% (28/94) with other communicable disease conditions and 13.8% (13/94) with non-communicable conditions (11 with clearly defined NCDs, the majority of which were cancer) (Table 2).

**Table 1:** Study group descriptives for all patients and those with culture-confirmed TB

	Within all patients (*n* = 94)		Within culture-confirmed TB cases (*n* = 26)	
	Proportion	95% CI	Proportion	95% CI
Age in years (IQR)	28 (24–33)	NA	29 (26–33)	NA
HIV status*				
HIV infected	62/84 (73.8%)	[63.5–73.8%]	21/24 (87.5%)	[69.0–95.7%]
HIV uninfected	22/84 (26.2%)	[18.0–36.5%]	3/24 (13.3%)	[4.3–31.0%]
Pregnancy status				
Pregnant or <6 w post-natal	63/94 (67.1%)	[57.0–75.7%]	20/26 (76.9%)	[58.0–89.0%]
Not pregnant or post-natal	31/94 (33.0%)	[24.3–43.0%]	6/26 (23.1%)	[11.0–42.1%]
Treatment history†				
Previously treated	11/86 (12.8%)	[7.3–21.5%]	2/22 (9.1%)	[2.5–27.8%]
Not previously treated	75/86 (87.2%)	[78.5–92.7%]	20/22 (90.1%)	[72.1–97.5%]
Admission diagnosis				
Pneumonia	29/94 (30.9%)	[22.4–40.8%]	8/26 (30.8%)	[16.5–50.0%]
Suspected PTB	24/94 (25.5%)	[17.8–35.2%]	12/26 (42.3%)	[28.8–64.5%]
PID	11/94 (11.7%)	[6.6–19.8%]	2/26 (7.7%)	[2.1–24.1%]
Cancer	8/94 (8.5%)	[4.4–15.9%]	0/26 (0%)	[0–12.9%]
Sepsis	6/94 (6.4%)	[3.0–13.2%]	2/26 (7.7%)	[2.1–24.1%]
Malaria	4/94 (4.3%)	[1.7–10.4%]	1/26 (3.8%)	[0.7–19.0%]
EPTB	4/94 (4.3%)	[1.7–10.4%]	1/26 (3.8%)	[0.7–19.0%]
CCF	3/94 (3.2%)	[1.1–9.0%]	0/26 (0%)	[0–12.9%]
UTI	2/94 (2.1%)	[0.6–7.4%]	0/26 (0%)	[0–12.9%]
Pre-eclampsia	1/94 (1.1%)	[0.2–5.8%]	0/26 (0%)	[0–12.9%]
Puerperal psychosis	1/94 (1.1%)	[0.2–5.8%]	1/26 (3.8%)	[0.7–19.0%]
Vulval Filariasis	1/94 (1.1%)	[0.2–5.8%]	0/26 (0%)	[0–12.9%]

IQR, interquartile range; PID, pelvic inflammatory disease; EPTB, extrapulmonary tuberculosis; CCF, congestive cardiac failure; UTI, urinary tract infection.

* HIV status was available for 84 of 94 cases.

† Treatment history was unavailable for 8 cases.

**Table 2:** Univariate and multivariate risk factor analysis for culture-positive TB infection in obstetric and gynaecological inpatients

	Culture-positive TB		Univariate		Multivariate*	
Risk factor	Proportion	95% CI	OR [95% CI]	*P* =	OR [95% CI]	*P* =
HIV status						
HIV infected	33.9% (21/62)	[23.3–46.3%]	3.244 [0.861–12.22]	0.082	3.244 [0.861–12.22]	0.082
HIV uninfected	13.6% (3/19)	[5.5–37.6%]	–	–	–	–
Pregnancy status						
Pregnant or <6 w post-natal	31.7% (20/63)	[21.6–44%]	1.938 [0.687–5.466]	0.211	1.909 [0.649–5.612]	0.240
Not pregnant or post-natal	19.4% (6/31)	[9.2–36.3%]	–	–	–	–
Treatment history						
Previously treated	18.2% (2/11)	[5.1–47.7%]	0.611 [0.121–3.074]	0.550	0.471 [0.090–4.469]	0.373
Not previously treated	26.7% (20/75)	[18.0–37.6%]	–	–	–	–
Age group†			1.001 [0.944–1.060]	0.983	0.990 [0.925–1.060]	0.773
15–20 years	0% (0/7)	[0–35.4%]	–	–	–	–
21–25 years	27.3% (6/22)	[13.2–48.2%]	–	–	–	–
26–30 years	33.3% (12/36)	[20.2–49.7%]	–	–	–	–
31–35 years	42.9% (6/14)	[21.4–67.4%]	–	–	–	–
36–40 years	25% (2/8)	[7.1–59.1%]	–	–	–	–
≥41 years	0% (0/7)	[0–35.4%]	–	–	–	–

Data are n TB positive/n tested (%) [95% CI], odds ratios (ORs) and p values present results of binary logistic regression analysis.

* Multivariate analysis was controlled for effect of HIV.

† Age was analysed as a continuous variable but is displayed as grouped to illustrate the age distribution of TB cases.

### Performance of the Xpert® MTB/RIF assay for detecting pulmonary tuberculosis

Of 26 culture-confirmed TB cases, the Xpert® MTB/RIF assay detected 21 of 26 culture-confirmed cases giving a sensitivity of 80.8% [95% CI: 60.0–92.7%]. With respect to specificity, the assay identified 66 of 68 culture-negative samples, giving a specificity of 97.1% [95% CI: 88.8–99.5%] (Table 3). The Xpert® MTB/RIF assay was significantly more sensitive than microscopy (21/26 (80.8%) *vs*. 13/26 (50.0%), *P* = 0.02) and detected an additional eight culture-confirmed cases. Numbers were low, but stratification of Xpert® MTB/RIF assay performance by HIV status found the assay to be 100% sensitive and specific in HIV-negative patients, with possible (but not statistically significant) poorer performance in HIV-infected patients (Table 3). The Xpert® MTB/RIF assay detected no cases of rifampicin resistance, failing to detect one rifampicin-resistant strain identified by culture DST. Two monoresistant *Mycobacterium tuberculosis* strains were detected in total, one rifampicin and one ethambutol resistant. No MDR-TB cases were identified by culture DST analysis (data not shown). Three samples initially gave an error on the Xpert® MTB/RIF assay. These were repeated and found to be negative.

**Table 3:** Xpert® MTB/RIF assay performance compared with MGIT culture and smear microscopy

	Sensitivity (95% CI)	Specificity (95% CI)	PPV	NPV
Xpert® MTB/RIF assay versus MGIT				
All patients*	21/26 (80.8%) [60.0–92.7%]†	66/68 (97.1%) [88.8–99.5%]	91.3% [70.5–98.5%]	93.0% [83.7–97.4%]
HIV negative	3/3 (100%) [31.0–100%]	19/19 (100%) [79.1–100%]	100% [31.0–100%]	100% [79.1–100%]
HIV positive	17/21 (80.1%) [57.4–93.7%]‡	39/41 (95.1%) [82.2–99.2%]	89.5% [65.5–98.2%]	90.7% [76.9–97.0%]
Sputum smear microscopy versus MGIT				
All patients	13/26 (50.0%) [30.4–69.6%]†	66/66 (100%) [93.3–100%]	100% [71.7–100%]	84.0% [73.8–90.8%]
HIV negative	2/3 (66.7%) [12.5–98.2%]	19/19 (100%) [79.1–100%]	100% [19.8–100%]	95.0% [73.1–99.7%]
HIV positive	10/21 (47.6%) [26.4–69.7%]‡	41/41 (100%) [89.3–100%]	100% [65.5–100%]	78.8% (65.5–88.5%]

Data displayed as number correct/number tested, (%) [95% CI]; PPV, positive predictive value; NPV, negative predictive value.

* The Xpert® MTB/RIF assay gave repeat errors in 3 samples and is excluded from this analysis. Fisher's exact test:

† *P* = 0.02

‡ *P* = 0.03.

### Potential risk factors

TB prevalence within HIV-infected patients was nearly 3-fold higher than that in HIV-uninfected patients (33.9% (21/62) *vs*. 13.6% (3/19)), with an associated 3-fold increase in risk of TB infection amongst HIV-infected patients (OR 3.244 [95% CI 0.861–12.221]) (*P* = 0.082) (Table 2). Multivariate binary logistic regression analysis (adjusting for the effect of HIV) demonstrated that the risk of TB was unaffected by patient age (OR 0.990 [0.925–1.060], *P* = 0.773), previous treatment history (OR 0.471 [0.090–4.469], *P* = 0.927) or pregnancy (OR 1.909 [0.649–5.612], *P* = 0.240) (Table 2). 42.3% (11/26) of TB cases were detected in patients with no other reason for admission other than suspected TB (Table 1). 50% (13/26) of TB cases were detected in patients with evidence of comorbidity with other communicable diseases: eight cases of pneumonia, two cases of sepsis (one septic abortion and one case of puerperal sepsis in HIV/AIDS), two cases of pelvic inflammatory disease (likely bacterial infection) and one case of malaria. The two remaining cases included one patient with acute cephalopelvic disproportion with suspected abdominal TB and a patient with puerperal psychosis (Table 1).

## Discussion

The key findings of this study are that in inpatients admitted to the obstetrics and gynaecology wards at UTH, Lusaka, Zambia, (i) pulmonary TB is an important incidental cause of concomitant comorbidity to the obstetric or gynaecological condition necessitating admission, (ii) as an alternative to sputum microscopy and culture, the Xpert® MTB/RIF assay provides a sensitive, specific and rapid method for diagnosis of pulmonary TB, and (iii) comorbidity of TB with HIV, other communicable and non-communicable diseases was common. The main limitation of this study was the relatively small number of patients. We have previously shown that TB can be detected in up to 10% of patients capable of producing sputum who are not clinically suspected of having pulmonary TB (Bates *et al*. 2012). As we only recruited patients with a cough, our data may underestimate the true prevalence of TB amongst obstetric and gynaecological admissions. Low numbers impaired the evaluation of the Xpert® MTB/RIF assay to detect rifampicin resistance.

National TB programmes in high HIV/TB burden countries require data from different patient groups to inform on how best to incorporate the Xpert® MTB/RIF assay into their diagnostic algorithms. In obstetric and gynaecological inpatients, we report a sensitivity of 80.8% (95% CI: 60.0–92.7%) and a specificity of 97.1% (95% CI: 88.8–99.5%), which is comparable with previous findings from the internal medicine wards at our centre (O'Grady *et al*. 2012), and elsewhere (Chang *et al*. 2012). With the high throughput of obstetrics and gynaecology wards, the Xpert® MTB/RIF assay could be a useful tool to rapidly screen for TB during the opportunity that presents during the obstetric or gynaecological admission or consultation. It may also help distinguish TB from other co-infections and comorbidities and ensure prompt initiation of TB therapy, reducing the risk of transmission to the baby and other family members. The one case of rifampicin resistance detected by DST was missed by the Xpert® MTB/RIF assay. False-negative rifampicin resistance has been well documented, and larger studies within high MDR-TB prevalence countries are required to evaluate whether the manufacturer has successfully addressed this issue (O'Grady *et al*. 2012; Lawn *et al*. 2013).

The prevalence of HIV infection within our study population was 73.4%. Whilst our study sample was small and major conclusions of HIV prevalence cannot be drawn, this high HIV burden amongst obstetric and gynaecological inpatients with suspected TB is comparable to that seen amongst females on the adult general internal medicine wards at UTH (Bates *et al*. 2012; O'Grady *et al*. 2012). This focus of HIV-associated TB cases highlights the need for broader active case finding in asymptomatic and lower-risk patient groups with community-based studies in Zambia previously showing that as little as 43% of TB cases occur in patients who would be classically defined as TB suspects (Ayles *et al*. 2009). In our study, there was a trend for HIV infection to be linked with a threefold increase in risk of TB infection compared with a 2-fold increase in risk of TB infection amongst HIV-infected patients on the internal medicine wards (Bates *et al*. 2012). A large community-based study of antenatal attendants in South Africa also found TB rates to be 3-fold higher amongst HIV-infected women (Gounder *et al*. 2011). We did not detect any significant decrease in risk of TB infection, with increasing age as shown previously (Bates *et al*. 2012), but this may likely be due to the younger age profile of our study group: median age of 28 (IQR: 24–33) *vs*. 35 (IQR: 28–43) (Bates *et al*. 2012), Mann–Whitney *U*-test, *P* < 0.001).

One of the challenges at busy, high-turnover referral hospitals is the focus on addressing the immediate obstetric or gynaecological reason for admission, whilst concomitant comorbidities with chronic communicable diseases like TB can be easily overlooked (Marais *et al*. 2013). High levels of TB comorbidity with other communicable and non-communicable disease have been previously documented amongst general internal medicine inpatients at the University Teaching Hospital in Lusaka (Bates *et al*. 2012). We hence sought to assess to what degree TB cases presented as comorbidities on the obstetric and gynaecological wards. We documented five cases of TB comorbidity with infectious diseases including pelvic inflammatory disease (PID), bacterial sepsis and malaria. 11.7% (11/94) of the recruited patients had a clearly defined non-communicable disease (cancer and congestive cardiac failure), and active TB was not detected in these cases. We detected 20 cases of pulmonary TB within women who were either pregnant or <6 weeks post-natal. We did not conduct follow-up to assess the risk of neonatal TB infection in the women recruited, but one South African study detected TB in 16% of neonates born to mothers with suspected or proven TB (Bekker *et al*. 2012). The majority of neonatal admissions have feeding tubes inserted, which presents an excellent opportunity for TB screening without the need for additional invasive sampling, possibly using the Xpert® MTB/RIF assay, which has been demonstrated to perform well on gastric aspirate at our site (Bates *et al*. 2013).

## Conclusions

The Xpert® MTB/RIF assay provides a sensitive, specific and rapid method for diagnosis of TB in obstetric or gynaecological inpatients. Most of the obstetric admissions and during the study were for deliveries with a short duration of inpatient stay. Similarly, the gynaecology admissions were for miscarriages with a similar short stay. The high TB/HIV co-infection rates in SSA and presence of TB with HIV comorbidities now call for more proactive and rapid screening for pulmonary TB and HIV in obstetric and gynaecological inpatient facilities to provide more optimal and synergistic alignment of health care for communicable and non-communicable disorders.
